# Vulnerabilities in migrant live-in care arrangements for people with dementia: a comparative analysis of experts’ insights from Germany and Israel

**DOI:** 10.3389/fpsyt.2025.1485270

**Published:** 2025-02-28

**Authors:** Natalie Ulitsa, Anna Eva Nebowsky, Liat Ayalon, Mark Schweda, Milena von Kutzleben

**Affiliations:** ^1^ Louis and Gabi Weisfeld School of Social Work, Bar-Ilan University, Ramat Gan, Israel; ^2^ Division for Ethics in Medicine, Department for Health Services Research, School VI - Medicine and Health Sciences, Carl von Ossietzky Universität Oldenburg, Oldenburg, Germany; ^3^ Division for Prevention and Rehabilitation Research, Department for Health Services Research, School VI - Medicine and Health Sciences, Carl von Ossietzky Universität Oldenburg, Oldenburg, Germany

**Keywords:** vulnerability, dementia care, migrant caregivers, experts, triadic care arrangement

## Abstract

**Background:**

The global rise in dementia among older adults has led to an increased reliance on migrant live-in caregivers, particularly in countries like Germany and Israel. This triadic care arrangement, involving persons with dementia, their families, and migrant live-in caregivers, presents unique challenges and vulnerabilities. These vulnerabilities, deeply intertwined with ethical concerns, are shaped by the socio-cultural and legal contexts of each country. This study aims to explore these vulnerabilities through a comparative analysis of expert experiences in Germany and Israel.

**Method:**

A qualitative study was conducted using semi-structured interviews with 24 experts—14 from Israel and 10 from Germany—who have extensive experience in dementia care or migrant caregiving. The interviews were analyzed through qualitative content analysis, focusing on six dimensions of vulnerability: physical, psychological, relational/interpersonal, moral, socio-cultural-political-economic, and existential/spiritual.

**Results:**

The analysis revealed that all parties in the care triad—persons with dementia, migrant live-in caregivers, and family members—experience distinct yet interconnected vulnerabilities. These vulnerabilities are deeply entangled, manifesting in complex, interrelated ways both within each party and between the different parties in this triadic arrangement. The study also highlighted both similarities and differences in expert experiences between Germany and Israel, reflecting the unique socio-cultural and legal contexts of each country.

**Conclusions:**

The study underscores the multifaceted and interdependent nature of vulnerabilities in migrant live-in care arrangements for people with dementia. By comparing expert insights from Israel and Germany, the research highlights the critical role of national policies and cultural contexts in shaping these vulnerabilities, leading to distinct experiences and challenges in each country. Addressing these vulnerabilities is essential for improving the quality of care and the well-being of all parties involved in the triadic care arrangement.

## 1 Introduction

The phenomenon of demographic aging is closely linked with an increasing number of older individuals living with dementia who require care services. Currently, more than 55 million people worldwide are living with dementia, with nearly 10 million new cases emerging each year (1). This growing burden is both significant and concerning. In many Western countries, including Germany and Israel, a majority of people with dementia reside within their communities, receiving care primarily from family members, often supplemented by migrant live-in carers (2, 3), thereby creating a triadic care arrangement. Providing dementia care with the assistance of migrant live-in carers entails a multifaceted set of difficulties, possible conflicts, and vulnerabilities (4–7). Vulnerability is closely tied to ethical issues in migrant live-in care for people with dementia due to several key factors, e.g. *protection of vulnerable groups* -ensuring the welfare, rights, and dignity of vulnerable populations, such as people with dementia and migrant caregivers, is a fundamental ethical concern; *risk of exploitation* - migrant caregivers face potential exploitation, and people with dementia risk neglect, highlighting the need for ethical vigilance and protective measures; *equity and justice* -the reliance on migrant caregivers reflects deeper societal inequalities, raising ethical questions about the fair distribution of care responsibilities and resources (8).

This research offers a comparative perspective on the complexities of this triadic care arrangement and explores vulnerabilities for all parties involved (persons with dementia, family members, and migrant live-in carers) based on the experiences of experts in migrant live-in dementia care in Israel and Germany.

In the following sections, we will provide a background on migrant live-in care in Israel and Germany, elaborate on the issue of vulnerabilities in aged care and home-based dementia care with migrant live-in carers, and explain the rationale and importance of the present study.

### 1.1 Migrant live-in care for people with dementia in Israel and Germany

Migrant live-in care for people with dementia plays a critical role in supporting older individuals in both Israel and Germany. Despite many similarities and common challenges across nations, notable differences are primarily influenced by each country’s unique geographical and socio-cultural contexts and legal frameworks. In Israel, regulated by the Long-Term Care Insurance Program (LTIP) since 1988, the system promotes ‘aging in place’ through home-based care. Israel has over 73,633 migrant caregivers—59,254 documented and 14,379 undocumented—origating from countries like the Philippines, India, Sri Lanka, Uzbekistan, Moldova, and Ukraine (9). Recent reforms since 2018 have enhanced the flexibility of care options and facilitated the employment of migrant live-in carers (10, 11).

In contrast, Germany’s care sector, which relies on about 500,000 Eastern European caregivers, operates within a less regulated ‘grey’ market (12, 13). German families often engage caregivers through agencies that navigate strict employment laws, facilitating frequent caregiver rotations. This practice, influenced by legal constraints and caregivers’ preferences due to their geographical proximity to their home countries, allows for more flexible employment arrangements but leads to less stable care relationships compared to Israel’s more regulated approach (6). These differences in regulatory frameworks and policies significantly impact the experiences and challenges faced by migrant live-in carers, people with dementia, and their families.

### 1.2 Vulnerabilities in migrant home-based dementia care

Migrant live-in care arrangements for people with dementia involve complex interactions between care recipients, migrant live-in carers, and family members, leading to various challenges, potential moral conflicts, and vulnerabilities (14–16). Building upon the initial exploration of challenges and vulnerabilities in migrant live-in dementia care, it is essential to delve deeper into the concept of vulnerability itself. Vulnerability, as analyzed in various studies, is a multifaceted concept that encompasses different types, definitions, and categories (17–20). Basic human vulnerability refers to the inherent condition affecting all individuals due to their human nature, characterized by the universal experience of ‘human finitude’ and susceptibility to harm and injury (21, 22). Various approaches to the concept of vulnerability agree that while we all share a common vulnerability, this vulnerability is distributed differently across individuals. Universal vulnerability can become exacerbated in certain social, political, and other contexts (23). Specifically, situational vulnerability arises from specific external conditions—such as cultural, social, political, and economic factors—that render some individuals more susceptible to harm than others (24).

In the context of aged care, Sanchini and colleagues (25), based on the latest studies, proposed six dimensions of vulnerabilities for older people that characterize aged care. *Physical vulnerability* is observed in the bodily deterioration associated with aging, which can lead to conditions like frailty, illness, dementia, and disability. *Psychological vulnerability* encompasses mental health changes, diminishing intellectual functioning, and emotional factors such as the cumulative loss of loved ones and the absence of emotional support. *Relational/interpersonal* vulnerability highlights the impact of human interdependence and the potential for conflicts and miscommunications. *Moral vulnerability* concerns ethical dilemmas, respect for dignity, and the potential for infantilization and depersonalization of older adults. *Socio-cultural, political, and economic vulnerabilities* reflect discrimination, economic instability, and marginalization faced by older adults and their caregivers. Lastly, *existential/spiritual vulnerability* pertains to existential questions about identity, purpose, and finitude experienced more intensely by older adults. These dimensions also offer insights into the vulnerabilities of persons with dementia in home-based care. However, it is imperative to explore the vulnerabilities of all parties involved in the triadic care arrangement—persons with dementia, family members, and migrant live-in caregivers—to fully capture the scope of challenges they face. This comprehensive approach will provide a holistic understanding of the care arrangement and the dynamics within it.

### 1.3 Present study: rationale and research questions

This study focuses on the vulnerabilities in dementia home care with migrant live-in carers. Addressing these vulnerabilities is crucial for enhancing the well-being and safety of everyone in the care triad. These vulnerabilities can significantly impact the quality of care for persons with dementia. Understanding them, especially through experts’ experiences, will help identify systemic inequities and gaps, leading to more effective support systems and policies that improve conditions for migrant live-in carers, persons with dementia, and their families.

Experts provide comprehensive insights into these vulnerabilities, drawing on a broad spectrum of experiences and observations. This is especially valuable given the practical challenges of directly accessing the care triad, whose members may face barriers to participation due to privacy concerns, health issues, or legal constraints. Furthermore, as experts positioned within intermediate structures such as organizational, community, and policy frameworks (meso-level), they offer invaluable insights that bridge the micro-level of individual experiences and the macro-level of national policies.

In this study, we obtained information from interviews with experts in Israel and Germany. We focus on Germany and Israel due to their aging populations, high life expectancy, and increasing numbers of people with dementia. Both countries have national dementia plans (Israel, 2013; Germany, 2020) and a growing number of migrant caregivers (10, 26). Despite similarities as modern Western countries, they also differ culturally (e.g., Israel is more collectivistic, with closer family ties and greater reliance on groups (27) and geographically. These differences in socio-cultural and geographic settings impact the legal and practical aspects of migrant live-in care arrangements. For example, in Israel, migrant live-in caregivers reside with the person with dementia for extended periods, while in Germany, live-in caregivers typically stay for 2-3 months and are rotated. Focusing on experts’ experiences in these two countries may help identify common and unique vulnerabilities and deepen our understanding of the influence of different socio-cultural and policy contexts on these vulnerabilities. In addition, the focus of most existing studies on single-country contexts (7, 28–30) constrains our comprehension of how diverse cultural and policy environments affect the nature and scope of these vulnerabilities. Our research seeks to bridge these gaps through a comparative analysis between countries, aiming to enhance our understanding of vulnerabilities in home-based care settings across different cultural and policy backgrounds.

In this study, we aimed to answer two research questions:

1. According to experts’ experiences, what are the vulnerabilities of each party involved in triadic arrangements (person with dementia, migrant live-in caregiver, family member)?
2. What are the commonalities and differences in experts’ experiences regarding vulnerabilities inherent in migrant live-in care arrangements for people with dementia between Israel and Germany?

## 2 Materials and methods

### 2.1 Study design

A qualitative methodology using semi-structured interviews was adopted to elicit experts’ experiences regarding complexities and vulnerabilities in home-based migrant care arrangements for older people with dementia. The study protocol was approved by the Bar-Ilan University (062201, June 2022) and Carl von Ossietzky Universität Oldenburg Ethics Committees (2022–049).

### 2.2 Sample composition and recruitment

This study included 14 Israeli and 10 German participants. We defined “experts” as individuals meeting the following inclusion criteria: 1) holding a senior position in a governmental, political, or public setting; 2) having substantial knowledge and experience in the field of dementia care or migrant home care for older people/people with dementia, and 3) Being directly involved in the recruitment and monitoring of migrant caregivers, including representatives of placement agencies, care and welfare organizations, NGOs, human rights organizations, and governmental structures. For this study, we excluded individuals who: 1) do not have substantial experience in dementia care or migrant home care, and 2) hold positions that do not provide direct insight or influence in the field of dementia or migrant care. For more detailed information regarding the characteristics of the experts in Germany and Israel, see **Table 1**.

**Table 1:** Characteristics of expert in Israel and Germany.

	Germany(n=10)	Israel(n=14)
**Gender** (% female)	40 %	86%
**Professional background**		
Representatives of Placement agencies for migrant live-in caregivers	5	0
Social Workers in a senior position in medical organizations/ Organizations for dementia care	1	2
Lawyers	1	2
Journalists (in the field of aging and old age)	0	1
NGO representatives	1	2
Politicians	2	0
Representatives from the Alzheimer’s association	0	2
Heads of departments in governmental organizations	0	2
Public figures (representatives/heads of communities)	0	1

Purposive sampling was used in both countries. In Israel, experts were recruited via researchers’ professional and personal connections. Additionally, targeted outreach was conducted through email to key persons in the field of dementia home-based migrant care to achieve diversity regarding their professional expertise and positions. In Germany, several strategies were used. Participants were primarily recruited through email, e.g., all directors of placement agencies. Some of the directors were chosen based on their agency’s size and status. Politicians were contacted based on their importance in the field of aged care and on variance in their political orientation/party affiliation.

### 2.3 Procedure

All participants signed informed consent sheets prior to participating in the study. In Israel, 14 semi-structured interviews with experts were conducted using *Zoom* or video telephone call platforms from October 2022 to June 2023. In Germany, ten semi-structured interviews with experts were conducted using the video call platform *Big Blue Button* between August 2022 and January 2023.

The interviews were conducted following a semi-structured interview guideline, which was developed jointly by the research teams in both countries in English and later translated into Hebrew and German. Interviews started by asking the participants to describe their professional background and experience with migrant live-in care arrangements for people with dementia. This was followed by questions aimed at gaining the expert’s views regarding dementia home-based care (e.g., “How do you perceive this form of care in comparison to other forms of care, for example, home? “). In this particular study, we focused on challenging situations, conflicts, and vulnerabilities, asking experts, for instance, the following questions: “Do you recognize problematic power structures within the arrangements? “; “Do you see potential conflicts in live-in care arrangements?”; “Can you describe vulnerabilities in live-in care arrangements?”; “In your opinion, is there a side that is more vulnerable in these triadic arrangements, and if so, which side?”. Interviews lasted an average of 60 minutes both in Israel and Germany and were conducted by members of the research teams with experience in qualitative research.

### 2.4 Data analysis

We employed a qualitative content analysis approach to ensure a thorough and nuanced examination of the expert interviews, following several steps outlined by Braun and Clark (31). The process began with verbatim transcription of the interviews, followed by multiple readings. Initially, guided by the study’s questions, we applied deductive coding to analyze expert interviews concerning the vulnerabilities of people with dementia, using a predefined set of categories based on the six dimensions of vulnerabilities in aged care identified in prior studies [e.g (32, 33)] and elaborated by Sanchini et al. (2022) (25). These dimensions (as mentioned in the introduction section) include: 1) physical, 2) psychological, 3) relational/interpersonal, 4) moral, 5) socio-cultural-political-economic, and 6) existential/spiritual. We used these six categories as a basis because they offer a comprehensive and up-to-date literature review-based view of the vulnerabilities of older people in need of care, including those with dementia. We chose to employ Sanchini’s and colleges’ framework (25), tailored initially to describe the vulnerabilities of older people in need of care and adapt it to all members of the triad because, to the best of our knowledge, no existing concept or model in eldercare comprehensively considers the vulnerabilities of all parties involved. This approach allowed us to systematically capture the multifaceted nature of vulnerabilities experienced by persons with dementia, their family members, and migrant live-in carers, providing a holistic understanding of the triadic care dynamics. For instance, the physical dimension of vulnerabilities for a person with dementia encompasses issues such as physical illness, cognitive decline or advanced dementia stages, increased frailty, and disability. In contrast, this dimension for migrant live-in caregivers might be expressed in physical and mental strain from caregiving tasks, including orthopedic problems, sleep deprivation, and the risk of workplace injuries. As for family members, this dimension of vulnerabilities may involve health issues and potential neglect of their own physical well-being.

This framework, detailing vulnerability dimensions for each party, served as the main categories for discussion within and across research teams in both countries until a consensus on the coding structure was achieved. The final phase involved identifying quotes/statements within the interview material that support these categories (dimensions of vulnerabilities) for each party involved in the care triad. Due to space constraints, we present a detailed description of each dimension of vulnerability for each party in the triadic home-based care arrangement in **Table 2**, and we provide examples of relevant quotes from the expert interviews in Israel and Germany in **Table 3**.

**Table 2:** Dimensions of vulnerabilities in dementia home care for persons with dementia, migrant live-in carers and family members.

Dimension	Person with dementia	Migrant live-in-care	Family member
Physical	Illness, cognitive deterioration, increased frailty, and disability.	Physical and mental strain from caregiving tasks (e.g., orthopedic issues, sleep deprivation), potential for workplace injury.	Health issues, potential for neglect of own physical health.
Psychological	Emotional distress, depression, anxiety, feelings of confusion, loneliness, psychological discomfort (due to invasion in a private space).	Stress, burnout, loneliness, and isolation due to cultural and language barriers.	Anxiety, guilt, and emotional strain from caregiving responsibilities or decision-making.
Relational/Interpersonal	Reduced social interactions, dependency on caregivers and family members, and potential isolation.	Challenges in establishing trusting relationships due to cultural differences and potential for professional isolation.	Altered family dynamics, increased dependency on migrant caregivers, loss of control/power.
Moral	Risk of being undervalued or stigmatized, infantilization, depersonalization, deprivation of personal dignity, and ethical considerations in care decisions.	Navigating ethical dilemmas in care, balancing professional duties with personal values, depersonalization (objectivization), and stigmatization.	Concerns over the quality and ethics of care provided and managing care decisions, moral conflicts, and moral distress.
Socio-Cultural-Political-Economic	Ageism, risk of marginalization, and reduced access to resources.	Legal vulnerabilities, discrimination, economic instability, and job insecurity; cross-cultural disparities, and language barriers,	Navigating healthcare systems, the financial burden of care, and societal expectations.
Existential/Spiritual	Facing existential questions about identity, purpose, and finitude.	Personal sacrifices, questioning life choices, and dealing with separation from own family.	Dealing with loss, grief, and existential concerns regarding the well-being of their loved one.

**Table 3:** Dimensions of vulnerabilities in triadic dementia home care arrangements accompanied by direct excerpts from experts’ interviews in Israel and Germany.

Dimension of Vulnerability	Person with Dementia		Migrant caregivers		Family members	
	Examples of quotations Israel	Examples of quotations Germany	Examples of quotations Israel	Examples of quotations Germany	Examples of quotations Israel	Examples of quotations Germany
**Physical**	"Dementia brings very negative consequences, greatly increasing the chance of negative outcomes in all areas of life, from health and functionality to cognitive decline, among others. (…) So yes, the older person is the weak link. In any case, I think the main victim is usually the older person with dementia because he is the weakest in this situation. I believe it's the older person who suffers the most because of this (dementia).”	“Or even months ago, so she had had a mild delirium and, according to her relatives, another severe episode of dementia. (...). The mother used to travel a lot. Since Corona, she's let many social contacts slide and no longer comes out of the house. He [the son of the Person with dementia] has now taken the car away because he has realized it's getting really risky. And now we're looking for a caregiver who has a driving license and who can clearly talk to the senior citizen and say, "Let's go shopping, let's go out and have a coffee" and get her going again”.	"I think it's scandalous that most foreign workers today are working 24/7. It's not humane, and then we're terribly shocked to find there's abuse and all sorts of things like that."	“The caregiver had tears in her eyes, slid a piece of paper over and said: Here one and twelve o'clock 3:15h 3:33 4:15 where she had to get up and couldn't catch up on sleep during the day.”	“Family members can harm themselves. They often neglect their own care, failing to visit doctors or address their health problems. This means there are associated risks with them being the main caregivers, including emotional, mental, physical, health-related, and financial risks.” “If it is a spouse who is the main caregiver for a person with dementia from among the family - he is also in an old age and may not be healthy, then he becomes even more vulnerable because in addition to his own health problems he has to care for his spouse”.	Not found
**Psychological**	“In the end, the introduction of a foreign caregiver into the home is an intrusion into the older person's private space by an outsider. This intrusion is often not easy, and we must also remember the position of the foreign caregiver, who also faces difficulties on their side.” “One of the greatest anxieties of older persons, as soon as a foreign caregiver enters the home, is the disconnection from the family. “(family member): It's okay, now we've made a vee, there's someone watching, and I can back off.” This anxiety ( to be alone without family members) can cause a lot of conflicts between the old man and the foreign caregiver”.	“No one wakes up in the morning and says to themselves, ‘I'm glad that a foreign Eastern European is moving in with me’. That's the big compromise that families make in order to avoid this stationary hell.”	“The vulnerability of the foreign caregiver arises often because they have to endure challenging behaviors from their care recipient, which is far from easy. I always remind myself, sitting in my chair as a social worker, telling her (the foreign caregiver), 'Don't take it personally, it's the disease speaking.' Yet, I constantly remember that there's a human being there, a person with feelings, with a heart, and someone who puts their all into their caregiving role, only to receive accusations like 'You stole from me,' 'You took from me,' 'Take your things and get out,' not to mention the instances of violence... Even if, five minutes later, the person with dementia settles down and stops making those accusations, it still creates a significant vulnerability for the foreign caregiver."	“She must then always, when the daughter comes or the son, is supposed to take herself back, officially, and act as if she is just working there and then sits in the kitchen and waits until they have drunk their coffee”	"Family members who are primary caregivers also represent a vulnerable population; they too are at risk because it becomes very difficult over time. Even if they are not directly involved in activities like bathing or cooking, they remain integral to the caregiving process. There's a constant sense of responsibility and obligation, which can be exhausting and wearing. This wear and tear, coupled with frustration and difficulty, can lead to a short path to causing harm." "Let's not ignore the underlying feeling, though it may be subtle, that here comes someone (live-in-care) who manages to take care of the mother, something that the three, four, or even seven of us (siblings) couldn't achieve."	“When the children are on site and are on the verge of burnout, so to speak, they are already so over it, over the day, over the bike, so completely hysterical. And then they want to save themselves by getting someone. And then it's just hysteria, hysteria, so on the outside it's like that and then it's over and then I wouldn't call it conflict anymore, but terrorism. So that's how it is when you see some children who have given themselves up to look after their parents.”
**Relational/interpersonal**	"Because there is a shortage of foreign caregivers today, you often find foreign caregivers with the power in their hands. They decide where to work, and if they don't feel good, they decide to leave or move to a place where they are paid more. This creates a kind of situation where the family has to keep the worker so he doesn't run away. To 'please' him... a gift here, a gift there, all kinds of conveniences. So maybe, just maybe, the fact that there is a shortage of caregivers gives them a lot of power in their hands."	"(…) So, the problem was, she somehow didn't think about things at the time or beforehand. And that was the problem. It was about millions. Her children, when they realized that the mother was no longer quite sane, let's say casually, they took over everything. Somehow, she was not incapacitated, but somehow, she had a care order. And so on. Somehow, they took over so they could empty the accounts."	“The foreign caregiver arrives, and he is a foreign worker in Israel (...)he is a foreign worker, there is no Israeli family here, the power is in the hands of the family. It is very hard…” “There are family members who may, and not only may, but they also do abuse the foreign live-in carer. They see them as a servant, someone who should be at the service of the older person, but also at their service. I have already heard of quite a few situations where the family hired the same live-in-care to do all sorts of things for them, including cleaning and other tasks. This is exploitation, abuse, inappropriate treatment, and disrespectful treatment towards that live-in by the family.” “Let's say there is a woman with dementia who has a partner. I have heard of situations where the partner attempted to initiate something with the live-in-care and not only attempted but also touched and did all kinds of things. So, maybe I understand his frustrations and shortcomings, but the live-in carer is not supposed to satisfy his emotional or sexual needs.”	“(...) I don't place cleaning ladies, we place caregivers. The service is housekeeping to a certain extent and on the other hand, of course, basic care. But I'm not allowed to go overboard here and then, let's say, misuse the staff by having the son-in-law say: "Oh, it's easy. Dad only has mild dementia and can stay alone for five hours. I pay €3,000 a month. Why don't you come over? I live three streets away and mow the lawn, let's be clear. We go this far and no further again and again.” “The others are really rootless here. Like truck drivers. They sit in their chambers and wait until they have to go back to work.”	“Loss of control, a genuine lack of control over what happens inside the house where the parent and caregiver reside. That's the reason why you hear about the use of cameras and other such measures.” “If there is a spouse living at home and there is a foreign caregiver living at home, a caregiver, it could be explosive, it is very difficult to bring a stranger into the home who would be an angel from heaven. It's not easy at all. absolutely not. You have a stranger at home. Not a guest, not a family member and this may create situations of tension, of conflicts, of discomfort, of unpleasantness”.	“But of course you already have a big dilemma on your hands. The family is of course beside themselves. That's understandable, they want you to leave immediately. They just want the person out of the house immediately. And they just have to see how they can manage it so that they still treat and see the person as a fully-fledged human being. And then simply bring everything back in an orderly fashion and, above all, offer the person assistance and simply not leave them to their own devices”
**Moral**	Not Found	"(…)So, and I was just a disruptive factor because I made sure that the mother could think more clearly again, because she had someone who took her seriously and who even started to talk to her about it, for example, to ask, somehow they made an order, because she was held like a convict. She somehow had no rights at all, she had fewer rights than anyone in any home, she was in a gilded cage but wasn't allowed out."	"The general public has a built-in lack of trust in foreign caregivers. I know... in courses I teach, when I would come to the topic of abuse and ask people, 'Who abuses an older person the most?' many times, the answer was - migrant caregivers." This caregiver spoke on behalf of her friends, also migrant caregivers who often say that they feel like a "slave" because they (families) treat them like slaves”. “They see her (a foreign caregiver) not as a human being, they sometimes see her as a caregiving tool that is inanimate, an inanimate tool “.	“I believe some [live-in carers] also would like to go to church on Sundays, have kind of rituals, but don’t dare to say that, because of course people [main carer/employer] say ‘But why, there is work’” “I have already experienced that a caregiver [live-in carer] called me and said: ‘Well, he is sitting on the toilet, his face is limp, what should I do now?’ I drove there and thought about calling the hospital on the way, but then I was only five minutes away and it was quite obvious that the customer had had a stroke and even the wife was standing next to him and said: ‘No, don't call an ambulance, he doesn't want to go to hospital’”	Not found	“Yes, money is money again. It's always about money somehow. (…). And I have two or three siblings who have dollar signs in their eyes, and one of them might say no, we promised Dad he'd stay in his house, and so on, and the others, no, go into a home and then you can sell the cottage.”
**Socio-cultural-political and economic**	"Misunderstandings due to language and communication difficulties are common, especially since many older people might not speak English well, if at all. They may struggle to understand the caregiver, and the caregiver might not understand them. This can lead to conflicts, even resulting in inappropriate or incorrect treatment (care). It's often the small things, like not responding to the person’s needs, that become problematic. Again, this is largely due to communication and language barriers.”	“Because of the language barrier. Then you have to see it this way, so purely from the point of view of the dementia patient, it's not so easy, ummm suddenly having another person in the apartment and uh that's not understandable to a certain extent and is sometimes seen as a foreign body that you have to get rid of, so that power struggles or conflicts arise, um, which can then escalate due to a lack of language barriers. So that doesn't necessarily go well, that's one thing, um so with the person themselves who is to be cared for and then of course there are also the cultural differences not only language differences but also cultural differences in other countries you sometimes have a completely different understanding of uh people with dementia and older people in general it depends very much on which culture the live ins come from, so the caregivers come from.”	"One foreign worker worked for an old woman who was wonderful in that she took her abroad; she went to Spain, Germany, and other places. The foreign caregiver really saw the world with this woman. But in the end, she decided to leave her because the woman always screamed at her and yelled at her. For her (live-in-care), culturally, it was a terrible thing to be yelled at; you don't yell like that in her culture. So, she gave up the job. These are the intercultural gaps, which I think are paid less attention to and are terribly important. The intercultural gaps are present in food and in the perception of what a family should look like, how care should be, and how relationships should be between people. Many times, families make a mistake because they extrapolate directly from Israeli culture to the relationship with the foreign caregiver, and they fail there because it is not the same. There is value in seeing the other culture. So, intercultural differences—this is one of the important things, starting with how her (foreign caregiver's) food smells. 'Ugh, disgusting,' is the reaction of the family. Stuff like that."	“We sometimes have very nasty senior citizens, but they are simply very difficult for us to deal with, and we realize ourselves that we usually ask them very, very honestly in the questionnaires that the relatives answer and say that they are verbally aggressive or that we have a big problem with some hidden National Socialism, I would say, that still lies dormant in their people. It's like this, come from Poland, it's always a bit more dangerous. But you only realize that when there is actually a care worker there all the time. And we immediately terminate anything that involves any kind of racism and say it's not possible.”	“This arrangement - (costs) is a lot of money. Many times, it is the family members who pay so it increases the vulnerability”. “I feel that when the family needs a foreign caregiver, they spend a lot of time getting the permit, which is a very, very important thing. It takes time - all the paperwork and bureaucracy”.	Not found
**Spiritual**	Not Found	Not found	Not found	Not Found	Not Found	Not found

## 3 Results

In exploring the vulnerabilities within migrant live-in care arrangements for people with dementia across Israel and Germany based on experts’ experiences, it becomes apparent that, according to them, all parties involved are vulnerable in different ways and that some of these vulnerabilities are interdependent.

In general, Israeli experts highlight the complex nature of vulnerability, suggesting that it is difficult to pinpoint the most vulnerable group within the care triad. While the person with dementia is often perceived in public opinion as the most vulnerable, Israeli experts acknowledged that each party in the triad—persons with dementia, migrant live-in carers, and family members (both spouses and children)—faces unique challenges that can amplify their respective vulnerabilities depending on the context and specific circumstances. In contrast, German experts focused more on the vulnerabilities of migrant live-in carers, drawing attention to their exposure to discrimination, excessive working hours, and challenging working conditions coupled with a lack of autonomy. Interestingly, the vulnerabilities of family members received relatively less attention in interviews with German experts, possibly reflecting their less extensive involvement in the caregiving process compared to their Israeli counterparts.

In the following, we present different dimensions of vulnerabilities that emerged from the analysis of interviews with experts in Israel and Germany, noting commonalities and specificities between the two countries.

### 3.1 Dimensions of vulnerabilities for persons with dementia, migrant caregivers, and family members

#### 3.1.1 Physical vulnerabilities

We identified various types of physical vulnerabilities that arise from the specific situations of each party in the caregiving triad. Israeli and German experts acknowledged these vulnerabilities for persons with dementia and migrant live-in carers, while only Israeli experts recognized them for family members. These vulnerabilities are partly interdependent and context-specific.

For persons with dementia, cognitive impairment significantly limits their physical and cognitive abilities, leading to considerable dependence on others and a diminished level of autonomy. This dependency is a primary reason for employing a migrant caregiver.

Migrant live-in carers, as acknowledged by both Israeli and German experts, face physical vulnerabilities resulting from the nature of their work, which includes managing the physical and behavioral symptoms of dementia. This can lead to chronic sleep deprivation, strenuous physical labor, and potential trauma from aggressive behaviors exhibited by older individuals with dementia.

Family members, even without direct physical involvement, may experience indirect physical vulnerabilities due to the caregiving burden. The physical and cognitive condition of their loved ones, coupled with the responsibility of coordinating care, can result in neglecting their own health, thus manifesting in a vulnerable physical state. However, it was observed that only Israeli experts, and not their German counterparts, highlighted the physical dimension of vulnerabilities among family members. This discrepancy could be influenced by cultural and geographical differences: for example, in Germany, a country much larger country in area than Israel, children often reside at a significant distance from their parents, resulting in less active involvement in caregiving. This geographical distance means that family members in Germany might not face the same physical strains of hands-on caregiving, potentially reducing their physical vulnerabilities. However, this can lead to other forms of vulnerability, such as emotional stress and anxiety, due to their inability to be physically present. In contrast, Israeli family members, who are more likely to live closer to their aging parents, are more actively involved in caregiving, which increases their physical vulnerabilities due to the direct physical demands and stresses of caregiving.

#### 3.1.2 Psychological vulnerabilities

Drawing from insights provided by experts in Israel and Germany, we identified several types of psychological vulnerabilities affecting all parties within the caregiving triad. Similar to the previous dimension, these vulnerabilities stem from the unique circumstances each party faces and are often interrelated.

For persons with dementia, experts from both Germany and Israel noted that the discomfort of welcoming a foreign caregiver into their home can lead to emotional stress and feelings of intrusion into their personal space, as well as an increased awareness of their dependency.

Migrant live-in-carers face psychological vulnerabilities resulting from being in a stranger’s private space in a foreign country and adapting to an unfamiliar culture. They might experience additional emotional stress due to separation from their families and being out of their comfort zone. German and Israeli experts both highlighted the psychological harm migrant caregivers may suffer. For instance, Israeli experts noted unfounded accusations of theft or violence from the person with dementia they care for, while German experts observed feelings of being belittled due to their status.

Family members also experience psychological vulnerabilities. The psychological strain of caregiving was recognized by experts in both Germany and Israel. Additionally, Israeli experts pointed out the complex emotions family members might experience, such as guilt and jealousy, due to hiring foreign caregivers, reflecting on their perceived inadequacies in providing care.

#### 3.1.3 Relational/interpersonal vulnerabilities

This dimension focuses on human interdependence, resulting in vulnerabilities. For individuals with dementia, their condition necessitates reliance on migrant caregivers and family members, who then overtly or covertly take up decision-making roles. Israeli experts have noted that due to a scarcity of migrant caregivers, these caregivers gain disproportionate power and may abruptly leave the person with dementia, possibly without notice, if they find better pay elsewhere. German experts emphasized the loss of autonomy and the dependence of a person with dementia on their adult children, who can sometimes abuse this power, leading to moral vulnerability, which will be described in the next section. This paternalism on the part of the children, which may stem from genuine concern or from a belief that a parent has lost the capacity to make decisions, can result in the denial of rights and a lack of consideration, turning the person into a “prisoner in their own house.”

Concerning migrant live-in carers, their relational/interpersonal vulnerabilities are linked to complex relationships with family members who inherently hold more control and power, potential exploitation and even sexual abuse, and loneliness stemming from being in an unfamiliar environment. Both Israeli and German experts acknowledged these issues.

Regarding family members, both Israeli and German experts highlighted the loss of control that adult children experience over what happens inside the house. Israeli experts noted that this has led to the adoption of surveillance cameras to monitor caregiving, while German experts reported cases of migrant live-in carers engaging in inappropriate behaviors, such as excessive alcohol consumption, which initially went unnoticed by relatives. This situation places family members in a moral quandary, as they feel compelled to protect the rights of the live-in caregiver, despite any misconduct, rather than terminating their employment hastily. Additionally, Israeli experts pointed out spouses’ discomfort with entrusting their homes to an “outsider,” which can also be challenging for them. These observations reflect the complex dynamics of trust, control, and vulnerability that characterize the caregiving relationship.

#### 3.1.4 Moral vulnerabilities

This dimension encompasses vulnerabilities within live-in care arrangements that are tied to overarching norms and values. These might be expressed as the risk of being stigmatized and undervalued for persons with dementia and migrant live-in carers, ethical dilemmas in care for migrant live-in carers, and family members’ concerns over ethics and quality of care for their loved ones.

For migrant live-in carers, Israeli experts describe depersonalization and their treatment by family members not as human beings but as tools to achieve a goal—referred to as the “objectification” of live-in carers or treatment of them as “slaves.” Furthermore, influenced by portrayals in public media regarding evidence of abuse of older people by migrant caregivers, live-in carers in Israel may experience public stigmatization and a built-in lack of trust from society, including family members and older persons- recipients of care. In Germany, experts highlight the moral dilemmas faced by live-in carers, who struggle to take time off due to the constant demands of their responsibilities, whether caring for a person with dementia or managing household tasks. In emergency situations, these caregivers must make rapid decisions about the health of the person with dementia, balancing not only the wishes of the individual but also those of the family members.

For persons with dementia, German experts point out specific moral vulnerabilities for them. They noted that cognitive decline and dependence of the person lead to their devaluation by family members, financial exploitation, deprivation of rights, and inability to take part in decisions regarding their own care.

For family members, German experts noted moral vulnerabilities that might arise when several siblings are involved. Financial disagreements between them may lead to ethical concerns and dilemmas about whether the parent’s funds should be viewed as a potential inheritance for them (the children) or if they should be allocated toward care expenses, such as employing a migrant live-in carer.

Notably, Israeli experts did not mention these particular moral vulnerabilities concerning persons with dementia or their family members.

#### 3.1.5 Socio-cultural, political, and economic vulnerabilities

These vulnerabilities refer to the risk of marginalization and reduced access to resources for persons with dementia; discrimination, economic instability, and job insecurity for migrant live-in carers; and the financial burden of care and societal expectations for family members. Both Israeli and German experts identified language barriers and cultural disparities between the person with dementia and the live-in carer as sources of clashes, misunderstandings, and conflicts that may lead to such vulnerabilities in the caregiving setting. Accounts range from persons with dementia feeling estranged in their own homes to the neglect of their needs and even power struggles that may escalate, leaving both parties feeling disregarded. The inability of persons with dementia to effectively communicate their needs and the inability of migrant live-in carers to understand and respond to these needs, coupled with existential interdependence, renders both parties vulnerable. Experts in both Israel and Germany also stressed that cultural differences contribute to these vulnerabilities. Live-in carers may feel unwelcome or even harassed due to these cultural differences. German experts specifically addressed covert racist attitudes toward Polish live-in carers from the care recipient’s side.

Regarding family members, German experts, except for indirectly mentioning workload, do not explicitly address vulnerabilities. However, Israeli experts recognize the financial constraints and bureaucratic challenges faced by family members as significant vulnerabilities.

#### 3.1.6 Spiritual vulnerabilities

This dimension remained unaddressed by experts in both countries in our study.

## 4 Discussion

This study aimed to comprehensively understand the vulnerabilities within the triad of dementia home-based care with migrant live-in caregivers, focusing on persons with dementia, live-in caregivers, and family members based on experts’ experiences in Israel and Germany. The relationships in home care arrangements with migrant live-in caregivers are complex and characterized by significant interdependence; each member of this triad relies on the others for their well-being in crucial ways (34). The exploration of vulnerabilities within this triadic setting, based on interviews with experts from Israel and Germany, reveals the multifaceted nature of this caregiving environment and its dynamics.

The complexities of vulnerabilities within the care triad were widely acknowledged. Israeli experts emphasized the intricate nature of these vulnerabilities. Contrary to the popular opinion among the general public and professionals, which often views the person with dementia as the most vulnerable member of the triad due to their health and mental condition (35), Israeli experts did not identify any particular side of the triad as the most vulnerable. Instead, they noted that vulnerabilities are present in all parties involved, stemming from their unique situations. Each party faces distinct challenges that can increase their vulnerability in specific contexts, making these vulnerabilities inherent to the triadic care arrangement. This aligns with existing literature that acknowledges different conditions leading to vulnerabilities: asymmetrical power relations and the intersection of ethnicity, culture, class, and legal status for migrant care workers; the poor physical and cognitive condition of people with dementia; and the emotional and physical burden experienced by family members (28, 36–38). However, German experts placed significant emphasis on the vulnerability of migrant live-in caregivers, highlighting their susceptibility to discrimination, excessive working hours, and challenging conditions.

The relatively lesser focus on family members’ vulnerabilities in Germany may be influenced by several socio-cultural factors, such as fewer children per family, greater geographic distance from parents, and more remote involvement in caregiving. These factors suggest a divergence in familial engagement between the two countries. However, it is important to note that these interpretations are derived from our analysis and were not explicitly probed during the interviews. Explicitly addressing this question with the experts could have provided deeper insights into these dynamics, and we recommend this for future research. Another explanation may be the different expectations in the two countries regarding family involvement in care, as Israel’s more collectivistic culture involves closer family ties (27), leading to greater family involvement in care.

In general, five of the six dimensions, except for spiritual vulnerability, were acknowledged by Israeli and German experts as relevant to all parties involved in triadic dementia home care arrangements. However, these vulnerabilities differ for each party according to their specific situations. While physical and psychological vulnerabilities are universally recognized, the emphasis on relational and moral vulnerabilities varies. Israeli experts noted the power dynamics and potential exploitation of migrant caregivers within the caregiving arrangement. This observation aligns with recent studies highlighting how relationships within the care triad can sometimes be discriminatory, reflecting power imbalances and the vulnerability of migrant live-in caregivers (7, 10). In contrast, German experts highlighted the moral dilemmas and decision-making challenges faced by migrant caregivers, underscoring the ethical complexities inherent in caregiving. Interestingly, the moral vulnerability dimension for a person with dementia was acknowledged by German experts but not by Israeli experts, potentially indicating a greater awareness in Germany of preserving the autonomy of people with dementia and probably a lower level of public stigma surrounding the condition. Supporting this, a study found that only 4% of the German population over the age of 50 reported fear of people with dementia, while over 80% expressed no fear, indicating relatively low levels of stigma in Germany (39). This could also stem from the more autonomy-oriented orientation of German society (27, 40).

Our findings also indicate a gap in addressing existential and spiritual vulnerabilities, suggesting that these aspects are often overshadowed by more immediate practical and ethical concerns. This oversight points to a potential area for further research and intervention, recognizing that spiritual well-being significantly impacts the quality of life for all parties involved (41).

In a comparative view, our analysis also showed that home care arrangements for people with dementia, along with the complex vulnerabilities for all parties involved, are significantly influenced by the legal policies specific to each country. These policies distinctly shape the vulnerabilities experienced by each party. For example, in Israel, a shortage of caregivers allows them to switch families for better pay, leading to concerns about caregivers gaining disproportionate power and potentially leaving their positions abruptly. Conversely, in Germany, while family members may wish to quickly dismiss a live-in caregiver for inappropriate behavior or keep them longer, they are constrained by legal policies requiring caregivers to rotate every three months. Such differing policies highlight the variations in how care arrangements are managed across these countries, underlining the distinct vulnerabilities that arise in Israel and Germany.

### 4.1 Entangled vulnerabilities in dementia care triads

This study aimed to deepen our understanding of the various vulnerabilities present in home care arrangements for people with dementia involving migrant live-in caregivers. We introduced a theoretical framework that distinguishes between different dimensions of vulnerability to address the challenges faced by each side of the triadic care relationship. However, the complex reality of caregiving—where individuals with varying health conditions, economic and legal statuses, cultural backgrounds, and generational differences interact within intricate human relationships—often results in these vulnerabilities becoming entangled, complicated, and interrelated. While previous studies have acknowledged the existence of vulnerabilities within home care arrangements, they typically addressed these vulnerabilities in isolation for each party involved (5, 42–44). Based on our findings, we propose viewing these vulnerabilities as entangled, interconnected, and interdependent rather than separate, highlighting the need for a more holistic approach to understanding and addressing them.

Interrelations *within a single party* of the triad refer to how different dimensions of vulnerability intersect and reinforce each other. For instance, our findings indicate that the “relational/interpersonal dimension of vulnerability” for a person with dementia can intensify their moral vulnerability. As German experts highlighted, when a person with dementia becomes increasingly dependent on others, they may lose autonomy, leading to a sense of diminished dignity. Similarly, Israeli experts revealed that the psychological vulnerability of family members, burdened by the responsibilities of caring for a parent with dementia and managing the relationship with a foreign caregiver, can manifest in physical vulnerability, such as neglecting their own health due to caregiving stress.

The interrelations of vulnerabilities *between the parties* of the triad highlight the entangled dependencies within the caregiving arrangement. For example, the physical vulnerability of a person with dementia, exacerbated by rapid health deterioration, can lead to increased physical or psychological strain on migrant live-in caregivers. These caregivers may face more demanding physical care tasks or suffer from sleep deprivation due to nighttime caregiving, leading to stress and burnout. This situation, in turn, can heighten the vulnerabilities of family members, who may experience increased stress, greater dependency on the caregiver, and concerns over care decisions, such as whether to continue with live-in care or opt for institutional care. Moreover, the socio-cultural, political, and economic vulnerabilities of migrant caregivers—who often occupy a lower position in terms of resources and power—are intricately linked to the vulnerabilities of family members, who bear the financial burden of employing a migrant caregiver. Language barriers, a form of psychological vulnerability, further complicate communication between all parties, leading to misunderstandings that affect the quality of care. For example, when people with dementia struggle to express their needs due to language differences, the caregiver’s ability to provide appropriate care is compromised, causing emotional stress and concerns over care quality among family members. In another example, the physical and cognitive decline of a person with dementia can create moral dilemmas for family members, who may face difficult decisions regarding care budgets and sibling relationships. These moral vulnerabilities can, in turn, influence the economic vulnerabilities of live-in caregivers, who might experience job insecurity based on the family’s decisions.

## 5 Conclusions

In summarizing our study, we can conclude that our research revealed multifaceted and interrelated vulnerabilities in dementia care arrangements with migrant live-in caregivers, illustrating the depth and complexity of the challenges faced by all parties involved in the triadic care arrangement. Furthermore, our findings emphasize the significant role of meso- and macro-level factors in shaping these vulnerabilities. By adopting a comparative research perspective, we have been able to identify how different socio-cultural and legal contexts influence the dynamics of these vulnerabilities.

For example, at the meso-level, the organizational structures within the care systems of Israel and Germany play a critical role in shaping the experiences of vulnerability for each party. In Israel, where migrant live-in caregivers reside with care recipients on a permanent basis, the constant presence of the caregiver can lead to a blurring of professional and personal boundaries. This close proximity might increase the relational vulnerability for both the caregiver and the care recipient, as tensions may arise from continuous interaction without sufficient breaks. Additionally, this setup can exacerbate the psychological vulnerability of caregivers due to the potential for burnout from being on call 24/7, while care recipients might feel a loss of privacy and autonomy in their own homes. In Germany, the less regulated grey market of migrant caregiving, where many caregivers are hired through agencies that navigate strict employment laws, creates a different set of challenges. The frequent rotation of caregivers, as required by German policies, disrupts the continuity of care, exacerbating the psychological vulnerability of both the person with dementia and the family members, who may struggle to build trust with constantly changing caregivers. This rotation system, while intended to protect caregivers from exploitation, can inadvertently lead to a lack of stability in care, highlighting how macro-level legal frameworks directly influence the relational and psychological vulnerabilities within the triad.

At the macro-level, broader socio-cultural and legal factors also play a pivotal role. For instance, Israel’s collectivistic culture, which emphasizes close family ties and a strong sense of responsibility toward older family members, often leads to higher involvement of family members in the caregiving process. This cultural expectation can heighten the physical and psychological vulnerabilities of family members, who may feel obligated to take on more significant caregiving responsibilities despite the presence of a live-in caregiver. In contrast, Germany’s more individualistic culture, where families are often geographically dispersed, reduces direct family involvement in daily caregiving tasks. While this can lessen the physical strain on family members, it can increase their psychological and emotional vulnerabilities due to feelings of guilt or helplessness when they cannot be physically present to care for their loved ones. This geographical and cultural distance can also create a sense of isolation for the person with dementia, as their primary emotional support system is not immediately available, further complicating their psychological and relational vulnerabilities.

These examples demonstrate how the interplay between meso- and macro-level factors, including organizational structures, legal frameworks, and cultural contexts, profoundly shapes the vulnerabilities experienced by each party in the triadic care arrangement. Understanding these complexities is essential for developing targeted interventions and policies that address the specific needs of each party involved, ultimately leading to improved care strategies in diverse socio-political environments.

## 6 Study limitations and strengths

The present study is not without its limitations. Firstly, the number of participants was relatively small, and the composition of the sample differed between Israel and Germany. However, these differences reflect the distinct organization of live-in care arrangements in each country. The German sample predominantly consisted of directors of placement agencies, who play a significant role in the migrant home care framework, while the Israeli sample included many social workers responsible for monitoring live-in care arrangements, as the employment of migrant live-in caregivers in Israel often relies on care recipients or family members.

Additionally, while the study mentions the country of origin of migrant caregivers, future studies could examine how the caregivers’ different cultural and socio-economic backgrounds influence the care dynamic, including vulnerabilities and resilience strategies within the triadic care arrangement.

Secondly, the findings of this study may not be generalizable to all settings or populations. Nevertheless, the comparative design allowed us to identify the influence of contextual factors, such as cultural and policy environments, on vulnerabilities in triadic care arrangements—insights that might not have emerged if the study had been conducted in only one country.

Thirdly, our study exclusively involved experts in the field, relying on their experiences as informants. While this is crucial for understanding the vulnerabilities of all parties from an intermediary perspective (between micro- and macro-levels), it is also a limitation. This choice was deliberate, as experts are uniquely positioned to synthesize diverse experiences and provide critical insights into systemic and policy-level complexities. To complement these findings, we have conducted interviews with individuals directly involved in triadic care arrangements across different countries. These interviews aim to provide a more direct and comprehensive perspective on vulnerabilities and care dynamics. We plan to publish these findings separately.

Despite its limitations, this study provides valuable insights into the complexities and vulnerabilities associated with migrant live-in care arrangements for people with dementia in Germany and Israel. Through comparative analysis, we identified both common and unique vulnerabilities within the caregiving triad, significantly shaped by the differing cultural and legal frameworks of the two countries. The proposed framework of vulnerability dimensions deepens our understanding of the challenges faced by each party, while the discussion of the interdependencies of these vulnerabilities’ sheds light on their deeply entangled nature.

While vulnerability is an ontological or universal condition inherent in human beings (21, 23), we strive to reduce these conditions as much as possible. Therefore, understanding these vulnerabilities within migrant live-in care settings is crucial for developing effective interventions that improve the well-being of all parties involved. This study contributes to the broader discourse on dementia care ethics and offers actionable insights for policymakers, care practitioners, and families, paving the way for improved care strategies in diverse socio-political contexts.

## Data Availability

Due to ethical considerations and participant confidentiality, the datasets from this study are not publicly accessible. Access to anonymized data may be granted upon request, pending approval from the relevant ethics committee.
